# Plasminogen Activator Inhibitor-1 and Vitamin D Association in the Overweight and Obese Pediatric Population

**DOI:** 10.3390/nu15173717

**Published:** 2023-08-25

**Authors:** Giovina Di Felice, Annamaria D’Alessandro, Anna Pastore, Michela Mariani, Danilo Fintini, Alessia Aureli, Simona Pezzi, Anna Lisa Montemari, Beatrice Barbara Rocco, Andrea Borseti, Andrea Onetti Muda, Melania Manco, Ottavia Porzio

**Affiliations:** 1 Clinical Laboratory Unit, IRCCS “Bambino Gesù” Children’s Hospital, 00165 Rome, Italy.; 2 Research Area of Management Innovations, Diagnostics and Clinical Pathways, IRCCS “Bambino Gesù” Children’s Hospital, 00165 Rome, Italy; 3 Endocrinology Unit, University Pediatric Clinical Department, IRCCS “Bambino Gesù” Children’s Hospital, 00165 Rome, Italy; 4 Research Unit of Multifactorial and Complexes Phenotype Diseases, IRCCS “Bambino Gesù” Children’s Hospital, 00165 Rome, Italy; 5 Department of Experimental Medicine, Tor Vergata University, 00133 Rome, Italy

**Keywords:** PAI-1, vitamin D, BMI, children, insulin resistance, HOMA-IR

## Abstract

Background: Childhood overweight and obesity have been described by the World Health Organization as noncommunicable diseases and among the greatest public health threats since they have reached epidemic proportions. A child with obesity risks becoming an adult with obesity and developing metabolic and hemostatic disorders which are the basis for the development of coronary heart diseases. Recently, a number of clinical reports have demonstrated that both an increase in plasminogen activator inhibitor-1 (PAI-1) and a deficiency in 25OH-vitamin D3 (VD) are associated with an increase in thrombotic episodes. Methods: PAI-1 and VD levels were measured in 259 clinically overweight and obese children aged between 2 and 18 years enrolled in the Nutritional Education Program of the Bambino Gesù Children’s Hospital and Research Institute of Rome (Italy) and 80 normal-weight subjects. Results: We observed increased HOMA-IR, PAI-1, and other inflammation indices associated with decreased VD levels when compared to normal-weight children. Conclusions: Our results demonstrated that overweight and obesity are correlated with higher levels of the inflammation index. Moreover, our patients show high PAI-1 and low VD levels, confirming the high thrombotic risk in our pediatric population.

## 1. Introduction

It has been demonstrated in adults that obesity is an important cause of chronic diseases such as hypertension, dyslipidemia, cardiovascular diseases, stroke, and diabetes [1]. Undeniably, an obese child may not only risk becoming an obese adult, but also risk developing metabolic and hemostatic disorders which are the basis for the development of coronary heart diseases [2,3]. Obesity is characterized by systemic inflammation; low-grade inflammation is triggered by inflammatory cytokines which are secreted by adipocytes and are responsible for macrophage activation in the adipose tissue [4].

Chronic inflammation triggers prothrombotic signaling pathways in vascular cells [5]. Indeed, the stimulation of vascular endothelium, platelets, and other cells by inflammatory cytokines leads to the over-regulation of procoagulant factors and adhesion molecules and to the down-regulation of anti-coagulant proteins, causing an increase in thrombin production and platelet activation [6].

Several studies have shown an association among obesity and hemostatic changes; obese subjects showed increased levels of plasminogen activator inhibitor-1 (PAI-1) [7,8].

Proinflammatory cytokines are also responsible for the increased hepatic and adipose release of anti-fibrinolytic agents, PAI-1, and tissue factor [9,10]. Plasminogen activator inhibitor type 1, a major regulator of fibrinolysis, is present in several tissue and cell types, including macrophages/monocytes, hepatocytes, vascular endothelium, adipose tissue of the heart and lungs, as well as in platelets [11,12]. This protein represents the main inhibitor of the plasminogen activation process in the blood and high levels contribute to thrombus formation and, consequently, to the onset and development of both acute and chronic cardiovascular diseases [13]. Plasma levels of PAI-1 are genetically regulated and are associated with risk factors for atherosclerosis such as hypertriglyceridemia, diabetes, and insulin resistance. The latter, indeed, characterized by obesity and the accumulation of visceral fat, plays a fundamental role in the regulation of PAI-1 gene expression. In vitro cellular studies show that insulin represents a potent inducer of PAI-1 synthesis in cells of hepatic origin and it acts through a complex mechanism of second messengers activation that have only recently been identified [14]. Despite numerous experimental evidence, the interpretation of clinical data does not provide a univocal explanation to attribute a direct role to insulin in the regulation of the circulating levels of PAI-1. Recently, it has been observed that even adipose tissue could contribute to the elevation of PAI-1 levels that are observed in a condition of insulin resistance.

There has been extensive research on the connection between hypovitaminosis D and obesity in the general population. A 2015 meta-analysis found an OR of 3.43 (95% CI: 2.33–5.06) between vitamin D (VD) insufficiency and obesity [15]. Due to metabolic processes that lead to the buildup of inactive forms, lower VD bioavailability, decreased tissue secretion, and decreased insulin sensitivity, VD insufficiency and excessive body fat have mutually harmful effects [15]. Why VD levels are lower in obese people is a subject of debate. The fundamental theory is that VD, which is fat soluble, is absorbed by adipose tissue [16]. In addition, due to inadequate sun exposure and a lack of outdoor activities, people with obesity produce less VD in their skin. Additionally, these people frequently eat junk food with low VD.

Recently, a number of clinical reports have demonstrated that VD deficiency is associated with an increase in thrombotic episodes, making evident the role of VD in the regulation of thrombosis-related pathways [17]. Therefore, the aims of this study are: (i) to determine the levels of PAI-1 protein in the pediatric age which will allow for the definition of a normal range which can be a useful tool for correctly estimating the presence of alterations of hemostasis; (ii) the correlation, if it exists, of BMI with VD levels in patients with obesity; and (iii) the study of some cardiovascular risk indicators and the possible correlation with both PAI-1 and VD levels.

## 2. Materials and Methods

### 2.1. Study Type

This cross-sectional study includes 259 consecutive patients with overweight (OW) or obesity (OB) enrolled in the Nutritional Education Program of the Bambino Gesù Children’s Hospital and Research Institute of Rome, Italy, and 80 normal-weight (NW) sex- and age-matched children referred to the outpatient clinic of the same hospital from October 2020 to February 2022.

The following criteria were used for patient (OW and OB) inclusion:

- Written informed consent from the parties who were legally able to do so, or from the parent(s) or legal guardian(s) of the child in accordance with local legislation;
- Consent for school-age subjects or subjects younger than 6 years old;
- Male or female subjects between the ages of 2 and 18;
- Overweight (i.e., a BMI between the 85th and 95th percentiles (1.064 SDS and 1.645 SDS, respectively) based on the Italian growth curves;
- Non-syndromic obesity (defined as a BMI below the 95th percentile and a BMI z-score for both age and sex below 1.064 SDS);
- Absence of endocrine or systemic disease (apart from obesity/overweight).

The following criteria were used for patient exclusion:

- No documented informed consent from the patient or parents or, if appropriate, no consent from the juvenile;
- Hereditary disorders such as genetic obesity and others;
- Any condition that is linked to a rise in inflammatory markers, especially values of erythrocyte sedimentation rate (ESR) ≥ 15 mm, C-reactive protein (CRP) ≥ 0.50 mg/dL, or a white blood cell count ≥ 16.00 × 10^3^/µL;
- Presence of cognitive deficiencies;
- Past or present thromboembolic or hemorrhagic episodes;
- The use of anticoagulant medication;
- The use of long-term corticosteroid treatment;
- A positive lupus anticoagulant (LA) test result;
- Previous bariatric surgery;
- Presence of allergies (i.e., total immunoglobulin E (IgE) ≥ 100.0 kU/L).

The following criteria were used for NW subject inclusion:

- Written informed consent from the parties who were legally able to do so or from the parent(s) or legal guardian(s) of the child in accordance with local legislation;
- Consent for school-age subjects or subjects younger than 6 years old;
- Male or female subjects between the ages of 2 and 18;
- A body mass index between the 10th and 84th percentiles was considered to be normal weight (BMI z-score for age and sex 1.064 SDS);

The following criteria were used for NW individual exclusion:

- No documented informed consent from the patient or parents or, if appropriate, no consent from the juvenile;
- Presence of inflammatory processes in the lower or upper respiratory tract, erythrocyte sedimentation rate values (ESR) ≥ 15 mm, C-Reactive Protein (CRP) ≥ 0.50 mg/dL, and number of white blood cells ≥ 16.00 × 10^3^/µL;
- Suspected celiac disease (anti-transglutaminase antibodies ≥ 20 CU);
- Past or present thromboembolic or hemorrhagic episodes;
- The use of anticoagulant medication;
- The use of long-term corticosteroid treatment;
- A positive lupus anticoagulant (LA) test result;
- Previous bariatric surgery;
- Presence of allergies (i.e., total immunoglobulin E (IgE) ≥ 100.0 kU/L).

### 2.2. PAI-1 Determinations

According to international recommendations, blood was drawn into vacutainer pediatric tubes (Greiner Bio-One, Kremsmünster, Austria), which had the same external dimensions as the adult ones but allowed the sampling of a small amount of blood (2 mL) with anticoagulant sodium citrate (0.109 M) at a ratio of 1:9 (*v*/*v*, sodium citrate: blood), centrifuged at 2000× *g* for 15 min at room temperature, and stored at −80 °C until use [18,19]. All blood samples were thawed in a water bath at 37 °C for a time period solely dedicated to thawing within 6 months of collection, homogenized by repeated moderate inversion, and quantified within 2 h. The PAI-1 contained in the test plasma forms a complex with the added urokinase, thus reducing the generation of plasmin from plasminogen. The quantity of generated plasmin (as measured by the pNA release at 405 nm) was measured. The STA-R Max2 coagulation analyzer (Diagnostica Stago, Asnières sur Seine, France) was used to examine the samples.

### 2.3. Insulin, Glucose, ALT, AST, CRP, Total Cholesterol, HDL, Triglycerides, and VD

Blood was collected in a serum tube (BD Advance 3.5 mL), centrifuged at 4000 rpm for 10 min at room temperature and analyzed with a COBAS 8000 instrument for clinical use (Roche Diagnostics S.p.A., Monza, Italy).

### 2.4. HbA1c, Leptin

Blood was collected in an EDTA K2E tube (BD 3 mL) and HbA1c was measured with the HPLC method (VARIANT II, Bio-Rad Laboratories, Hercules, CA, USA). Leptin was measured with the DRG Leptin Sandwich ELISA test (DRG Instruments GmbH, Marburg, Germany).

### 2.5. Platelets Count

Blood was collected in an EDTA K2E tube (BD 3 mL) and the platelets count was performed with the ADVIA 2120i analyzer (Siemens Healthcare Diagnostics S.r.l., Milano, Italy).

### 2.6. Statistical Analysis

The statistical analysis was carried out using the GraphPad Prism (version 9.0, GraphPad Software, San Diego, CA, USA). The data were checked to see if they had a normal distribution using both the histogram and the Kolmogorov–Smirnov test. When the data were normally distributed, parametric tests were applied, such as the *t*-test. A value of 0.05 was considered statistically significant in all statistical investigations, while a value of 0.01 was considered to be extremely statistically significant. PAI-1 and VD levels were characterized in the population using mean and standard deviation. The correlation between the clinical features collected for each study group, such as VD and BMI, was studied using the Spearman’s rank correlation analysis.

## 3. Results

The clinical and demographic information about the patients under study is described in Table 1. OB patients’ anthropometric and biochemical parameters were compared to those of NW and OW subjects.

The mean BMI value in our OB population was 32.2 (±6.4), with a mean age of about 12.5 years (±3.2), whereas our NW and OW patients showed a BMI of 18.1 ± 2.9 and 24.0 ± 2.6. Regarding cholesterol, although the OB total levels were between the reference values for the pediatric population and comparable to those of OW subjects (155.2 ± 27.5 vs. 148.2 ± 25 mg/dL; *p* = 0.093), they were higher than those with NW. The LDL form was instead significantly higher in both OW and OB compared to NW children (85.3 ± 22.0 and 95.4 ± 25.7 vs. 68.1 ± 16.4 mg/dL, respectively) and the HDL lower compared to NW subjects. Through using elevated fasting plasma LDL cholesterol, low HDL cholesterol, elevated blood glucose, and elevated insulin levels as risk factors, these findings support the association between obesity and cardiovascular risk [20].

In comparison to NW, triglycerides were also higher in OW and OB patients. Furthermore, we discovered higher HOMA-IR values in OB patients when compared to pediatric children without increased metabolic risk (4.3 vs. 1.9; *p* < 0.001) [21], confirming obesity as a risk factor for metabolic disorders [22,23].

**Figure 1:**
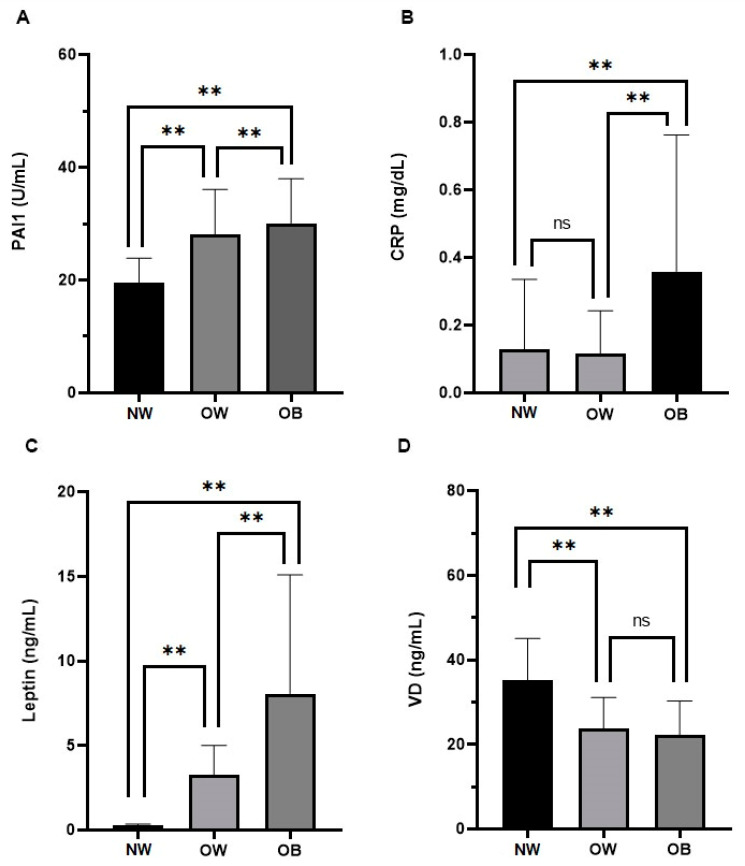
Comparisons of PAI-1 (**A**), CRP (**B**), leptin (**C**), and VD (**D**) levels in normal weight (NW), overweight (OW), and obese (OB) patients. ns = not significant; ** *p* < 0.01. All parameters are presented as mean ± standard deviation. Abbreviations: plasminogen activator inhibitor-1 (PAI-1); C-reactive protein (CRP); and vitamin D (VD).

As reported in Figure 1, PAI-1 levels were higher in OB compared to both OW and NW patients (*p* < 0.05; Panel A); PLT levels were also higher in OW and OB patients compared to NW subjects. As PLTs play a crucial role in coagulation, hemostasis, thrombosis, immunomodulatory processes, and inflammation [24], these results confirm the pivotal role of both PAI-1 and PLT in obesity-driven cardiovascular risk.

We found high WBC levels in our OB patients (*p* < 0.001), confirming the low-grade inflammation brought on by adipose tissue. Moreover, CRP was also higher in OB patients with respect to OW and NW individuals (*p* < 0.001; Panel B). Leptin levels were higher in both OW and OB patients when compared to NW subjects (*p* < 0.001; Panel C). Finally, VD levels were lower in both OW and OB patients compared to NW individuals (Panel D).

**Figure 2:**
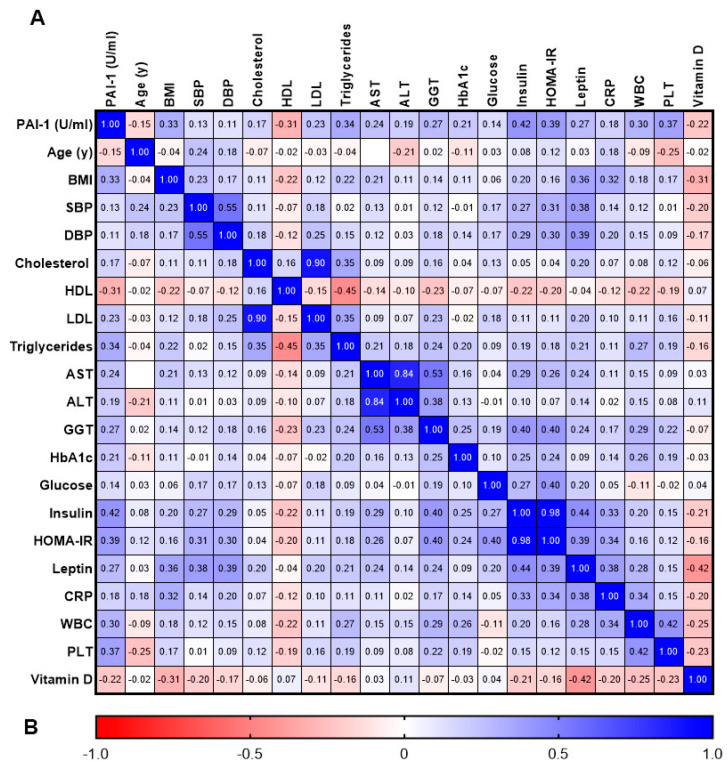
Correlations between PAI-1 and the other parameters studied. Values are expressed as Pearson correlation coefficient (r) (**A**). Color explanation is reported in (**B**), with the scale ranging from negative correlation (red) to positive correlation (blue). White box indicates no correlation.

In Figure 2, we report the correlation analysis of the parameter studied between the study groups. PAI-1 concentrations correlated positively with BMI (*p* < 0.001), LDL (*p* < 0.001), triglycerides (*p* < 0.001), AST (*p* < 0.001), GGT (*p* < 0.001), HbA1c (*p* < 0.001), insulin (*p* < 0.001), HOMA-IR (*p* < 0.001), and CRP (*p* < 0.001), and negatively with HDL (*p* < 0.001) and VD levels (*p* < 0.001). Moreover, VD levels correlated negatively with BMI (*p* < 0.001), PAI 1 (*p* < 0.001), leptin (*p* < 0.001), and other inflammatory indices studied.

## 4. Discussion

In our OB patients, we found significant changes in all of the markers studied compared to those of NW subjects. In particular, lipids (cholesterol, HDL, LDL, and triglycerides), hepatic enzymes (AST and GGT), metabolic markers (BMI, glucose, insulin, HbA1c, and HOMA-IR), inflammatory markers (WBC, PLT, CRP, and leptin), as well as PAI-1 and VD showed significant differences among NW and OB children. When compared to OB, OW subjects’ biochemical parameters were changed except for VD levels, indicating that the lowering of VD is an early phenomenon starting with the increase in the BMI. This is reinforced by the finding of lower VD levels in OW patients compared to NW patients.

It has been established that inflammation and obesity are correlated [25]. The transmigration of bone marrow-derived monocytes into adipose tissue is facilitated by leptin and other chemokines. As a result, levels of cytokines and acute-phase proteins, including CRP, rise, resulting in chronic low-grade inflammation [26,27]. This inflammation actively contributes to changes in hematologic parameters and affects the risk of thrombosis [23]. Additionally, platelets express the leptin receptor [24], and leptin potentiates platelet aggregation by agonists. In line with these earlier discoveries, we found that platelets and CRP levels were higher in our OB patients with respect to NW individuals.

The indication that obesity is a risk factor for metabolic disorders was further supported by the fact that we discovered raised HOMA-IR values in comparison to those reported for juvenile participants without increased metabolic risk [21]. These findings, which are supported by higher fasting plasma LDL cholesterol, lower HDL cholesterol, and higher levels of blood sugar and insulin, indicate the association between obesity and an increased risk of cardiovascular disease [22].

We also found high levels of WBC in our OB patients (*p* < 0.001). Our results agree with those recently reported [27] in which higher WBC levels in overweight and obese youngsters were found. Authors stated that this indicator could be used as an indicator of insulin resistance, and that low-grade inflammation brought on by adipose tissue has a deleterious impact on distal organ function through insulin resistance, which may be the cause of obesity-related problems.

The results of our investigation, which found a significant negative correlation between VD and leptin levels, corroborate those of a few other recent studies on adult cohorts [28,29,30] and pediatric subjects [31]. All these findings agree with our results, even if our investigation was unable to determine the underlying reason for the negative association, although there may be several factors at play. First, VD insufficiency has been linked to leptin resistance, which can result in hyper-leptinemia and insulin resistance. Second, VD deficiency has been related to the direct inhibitory action of VD on leptin release from adipose tissue [32]. It has been proposed earlier that alternative pathways may be more significant with progressive and persistent obesity as opposed to VD-mediated suppression of leptin secretion from adipocytes [33]. It is therefore necessary to determine the precise mechanism by which VD interacts with leptin release and plays a part in the in vivo regulation of leptin levels. Further research is then needed to fully understand this complex link and determine if hyper-leptinemia causes low VD levels or whether low VD levels cause hyper-leptinemia.

Blood stasis, changes in the vessel wall, and changes in the blood’s composition were Virchow’s three basic hypotheses for the causes of thrombosis [34]. While arterial thrombosis has been connected to endothelium disruption and platelet activation, venous thrombosis has been linked to hypercoagulability (composition change) and decreased blood flow (stasis) [35]. The conventional understanding that venous and arterial thrombosis are two distinct conditions has recently been questioned [36]. Patients who have arterial thrombosis run the risk of acquiring venous thrombosis and there are shared risk factors for both conditions [37]. Additionally, a number of other variables in the etiology of thrombosis have surfaced. One of the things that has proved to be an intriguing component implicated in regulatory mechanisms connected to thrombosis is the plasma levels of VD metabolites and their associated molecules [38]. Clinical reports have provided some tenuous evidence linking VD insufficiency to an increase in thrombotic events [39,40]. In our patients, lower levels of VD were found and this deficiency was correlated with BMI.

In addition, pro-inflammatory cytokines are also responsible for the increased hepatic and adipose release of anti-fibrinolytic agents, PAI-1, and tissue factor [9,10]. PAI-1, a major regulator of fibrinolysis, is present in several tissue and cell types, including macrophages/monocytes, hepatocytes, vascular endothelium, adipose tissue of the heart and lungs, as well as in platelets [11,12]. This protein represents the main inhibitor of the plasminogen activation process in the blood and high levels contribute to thrombus formation and, consequently, to the onset and development of both acute and chronic cardiovascular diseases [13]. As the main inhibitor of fibrinolysis, high levels of PAI-1 can increase coronary heart disease risk. Increased PAI-1 is involved with the control of insulin signaling in adipocytes. Insulin resistance (IR) has been associated with elevated PAI-1 levels and altered plasma lipids, which helps to explain the characteristic prothrombotic state of these pathologies [41]. Cura-Esquivel and collaborators recently reported that their overweight and obese children showed elevated PAI-1 levels compared with normal-weight subjects [41]. Those results agree with our findings, as we found significantly higher PAI-1 values in both OW and OB patients compared to NW children.

Our results indicate the rise of low-grade inflammation with the increase in BMI, low VD levels, and high PAI-1 concentrations in our OW and OB pediatric patients, thus confirming the risk of future thrombotic events in this population.

Our study has some limitations. First, because this is a cross-sectional study, we identified some correlations but were unable to determine a cause-and-effect connection. Second, as we did not analyze interleukins, we did not characterize the profile of both pro- and anti-inflammatory cytokines, failing to show their relationship with adipometrics parameters.

## 5. Conclusions

Taken together, our results demonstrate that overweight and obesity are correlated with higher levels of inflammation index. Moreover, our patients showed high PAI-1 and low VD levels, confirming the thrombotic risk in our pediatric population. However, as also sustained by the latest research [42], VD deficiency could be repaired by its supplementation, thus allowing a reasonable approach to prevent obesity complications. Furthermore, it was recently demonstrated that early dietary interventions and supplementation decrease chronic inflammation in children and adolescents [43]. Despite these efforts, children who are overweight or obese had slightly higher VD levels after taking VD supplements, and the impact on metabolic and cardiovascular outcomes is still debatable. There should be new initiatives to promote effective treatments to enhance the health of overweight and obese children and adolescents.

## Figures and Tables

**Table 1:** Clinical and demographic characteristics of our patients.

	Normal Weight	Overweight	Obese	*p*	*p*	*p*
	*n* = 80	*n* = 32	*n* = 227	(NW vs. OW)	(NW vs. OB)	(OW vs. OB)
Age, y, mean (standard deviation)	13.4 (3.2)	13.4 (2.7)	12.5 (3.2)	0.882	<0.05	<0.05
BMI, mean (standard deviation)	18.1 (2.9)	24.0 (2.6)	32.2 (6.4)	<0.001	<0.001	<0.001
SBP, mm Hg, mean (standard deviation)	109.1 (10.2)	115.9 (6.6)	120.6 (8.8)	<0.001	<0.001	<0.001
DBP, mm Hg, mean (standard deviation)	64.9 (9.3)	66.5 (5.3)	69.7 (8.8)	0.26	<0.001	0.001
Cholesterol, mg/dL, mean (standard deviation)	138.2 (23.7)	148.2 (25.0)	155.2 (27.5)	0.08	<0.01	0.093
HDL, mg/dL, mean (standard deviation)	62.7 (16.8)	54.0 (11.8)	47.3 (10.2)	0.051	<0.01	<0.01
LDL, mg/dL, mean (standard deviation)	68.1 (16.4)	85.3 (22.0)	95.4 (25.7)	<0.01	<0.001	<0.01
Triglycerides, mg/dL, median (5th and 95th percentile)	32.0 (30.0–112.5)	56.5 (32.0–111.5)	82.0 (45.0–196.4)	0.578	<0.001	<0.001
AST, U/L, median (5th and 95th percentile)	18.0 (8.1–34.0)	15.0 (8.0–33.5)	20.5 (11.0–61.0)	0.311	<0.001	<0.001
ALT, U/L, median (5th and 95th percentile)	44.0 (10.0–44.0)	20.0 (12.0–44.0)	21.0 (14.0–40.9)	0.132	0.197	<0.01
GGT, U/L, median (5th and 95th percentile)	7.0 (6.0–25.5)	11.0 (6.0–20.0)	15.0 (4.9–34.2)	0.611	<0.001	<0.001
HbA1c, mmol/mol, mean (standard deviation)	29.0 (1.5)	33.0 (3.1)	33.8 (3.8)	<0.001	<0.001	0.229
Glucose, mg/dL, mean (standard deviation)	85.3 (9.1)	83.7 (9.3)	87.5 (7.7)	0.394	0.074	<0.05
Insulin, U/mL, median (5th and 95th percentile)	9.9 (9.6–9.9)	11.4 (6.1–32.5)	20.4 (5.4–50.7)	<0.01	<0.001	<0.001
HOMA-IR, median (5th and 95th percentile)	1.9 (1.0–2.0)	2.1 (0.7–6.6)	4.3 (1.1–11.8)	<0.01	<0.001	<0.001
Leptin, ng/mL, median (5th and 95th percentile)	0.2 (0.2–0.3)	2.1 (0.2–7.5)	5.9 (2.0–23.0)	<0.001	<0.001	<0.001
CRP, mg/dL, median (5th and 95th percentile)	0.1 (0.0–0.8)	0.04 (0.03–0.79)	0.25 (0.34–1.09)	0.321	<0.01	<0.001
WBC, 10^3^/mL mean (standard deviation)	6.0 (2.2)	6.6 (1.4)	7.6 (1.8)	0.067	<0.001	<0.001
PLT, 10^3^/mL mean (standard deviation)	244.9 (79.9)	276.5 (55.7)	297.0 (68.8)	<0.05	<0.001	<0.05
PAI-1, U/mL, mean (standard deviation)	15.7 (0.9)	22.9 (5.5)	30.4 (7.9)	<0.001	<0.001	<0.001
VD, ng/mL, mean (standard deviation)	35.1 (9.9)	23.7 (7.4)	22.3 (8.0)	<0.001	<0.001	=0.43

Abbreviations: body mass index (BMI); systolic pressure (SYP); diastolic pressure (DIP); high-density lipoproteins (HDL); low-density lipoproteins (LDL); aspartate aminotransferase (AST); alanine aminotransferase (ALT); gamma glutamyl-transferase (GGT); glycated hemoglobin (HbA1c); homeostasis model-assessment-estimated insulin resistance (HOMA-IR); C-reactive protein (CRP); white blood cells (WBC); platelet (PLT); plasminogen activator inhibitor-1 (PAI-1); and vitamin D (VD).

## Data Availability

The authors confirm that the data supporting the findings of this study are available within the article. Raw data that support the findings of this study are available from the corresponding author, upon reasonable request. The data are not publicly available due to privacy and ethical reasons.
